# Supplementary material for: Meta-Analysis and Gene Set Enrichment Relative to ER Status Reveal Elevated Activity of MYC and E2F in the “Basal” Breast Cancer Subgroup
Source: PLoS One. 2009 Mar 9;4(3):e4710. doi: 10.1371/journal.pone.0004710 (PMC2650420; doi:10.1371/journal.pone.0004710)
Supplement: Dataset S1 — GSEA results from first screen. The file contains a zipped archive of the HTML results of the second GSEA screen. Reports for enriched or depleted datasets may be accessed from the “index.html” link within the my_analysis.GseaPreranked.1219975950625.rpt.zip ZIP archive. Enrichment plots are available for each of the 50 most enriched and depleted gene sets. (13.32 MB ZIP) [file pone.0004710.s001.zip › my_analysis.GseaPreranked.1219975950625.rpt.zip!//HDACI_COLON_CUR24HRS_DN.html]

Details for gene set HDACI\_COLON\_CUR24HRS\_DN[GSEA]

|  || Dataset | zSco.ERneg.to.ERpos\_collapsed |
| Phenotype | NoPhenotypeAvailable |
| Upregulated in class | na\_neg |
| GeneSet | HDACI\_COLON\_CUR24HRS\_DN |
| Enrichment Score (ES) | -0.5163699 |
| Normalized Enrichment Score (NES) | -1.5860023 |
| Nominal p-value | 0.023498695 |
| FDR q-value | 0.13308163 |
| FWER p-Value | 0.999 |
Table: GSEA Results Summary

  

Fig 1: Enrichment plot: HDACI\_COLON\_CUR24HRS\_DN      
 Profile of the Running ES Score & Positions of GeneSet Members on the Rank Ordered List

  

| PROBE | GENE SYMBOL | GENE\_TITLE | RANK IN GENE LIST | RANK METRIC SCORE | RUNNING ES | CORE ENRICHMENT || 1 | SLC39A14 |  |  | 1149 | 3.309 | 0.0077 | No |
| 2 | COL4A3 |  |  | 1335 | 3.098 | 0.0819 | No |
| 3 | PLEKHQ1 |  |  | 3225 | 1.827 | -0.0082 | No |
| 4 | MARCH3 |  |  | 5823 | 0.871 | -0.1787 | No |
| 5 | INHBA |  |  | 6164 | 0.762 | -0.1826 | No |
| 6 | MGAT2 |  |  | 8433 | -0.049 | -0.3516 | No |
| 7 | PPARG |  |  | 9735 | -0.688 | -0.4299 | No |
| 8 | THAP7 |  |  | 9831 | -0.745 | -0.4158 | No |
| 9 | TSPAN5 |  |  | 10584 | -1.231 | -0.4374 | No |
| 10 | SNX24 |  |  | 11636 | -2.152 | -0.4552 | Yes |
| 11 | IQWD1 |  |  | 11942 | -2.489 | -0.4074 | Yes |
| 12 | SPRY1 |  |  | 11974 | -2.533 | -0.3377 | Yes |
| 13 | TSNAX |  |  | 12040 | -2.628 | -0.2679 | Yes |
| 14 | FOSB |  |  | 12385 | -3.084 | -0.2060 | Yes |
| 15 | FOS |  |  | 12389 | -3.085 | -0.1186 | Yes |
| 16 | PPP2R5E |  |  | 12502 | -3.263 | -0.0342 | Yes |
| 17 | KIAA0143 |  |  | 12566 | -3.364 | 0.0567 | Yes |
Table: GSEA details [plain text format]

  

Fig 2: HDACI\_COLON\_CUR24HRS\_DN: Random ES distribution      
 Gene set null distribution of ES for **HDACI\_COLON\_CUR24HRS\_DN**
